# Tissue factor pathway inhibitor – cofactor-dependent regulation of the initiation of coagulation

**DOI:** 10.1097/MOH.0000000000000838

**Published:** 2024-08-26

**Authors:** Josefin Ahnström, Anastasis Petri, James T.B. Crawley

**Affiliations:** Centre for Haematology, Department of Immunology and Inflammation, Hammersmith Hospital Campus, Imperial College London, London, UK

**Keywords:** anticoagulant, factor V short, haemostasis, protein S, tissue factor pathway inhibitor

## Abstract

**Purpose of review:**

In humans, tissue factor pathway inhibitor (TFPI) exists in two alternatively spliced isoforms, TFPIα and TFPIβ. TFPIα consists of three Kunitz domains (K1, K2 and K3) and a highly basic C-terminal tail. K1 inhibits the tissue factor-activated factor VII complex, K2 specifically inhibits activated factor X, K3 is essential for interaction with its cofactor, protein S, and the basic C-terminus is binds factor V-short (FV-short) with high affinity. TFPIβ consists of K1 and K2 that is glycosylphosphatidylinositol anchored directly to cell surfaces. This review explores the structure/function of TFPI and its cofactors (protein S and FV-short), and the relative contributions that different TFPI isoforms may play in haemostatic control.

**Recent findings:**

Recent data have underscored the importance of TFPIα function and its reliance on its cofactors, protein S and FV-short, in influencing haemostatic control as well as bleeding and thrombotic risk

**Summary:**

TFPIα is likely the most important pool of TFPI in modifying the risk of thrombosis and bleeding. TFPIα forms a trimolecular complex with FV-short and protein S in plasma. FV-short expression levels control the circulating levels of TFPIα, whereas protein S exerts essential cofactor mediated augmentation of it anticoagulant function.

## INTRODUCTION

Haemostasis corresponds to the cellular and biochemical processes that limits bleeding following blood vessel damage. To be effective, the haemostatic response at the site of vessel injury must be rapid but also highly controlled to specifically localize clot formation – both spatially and temporally. Haemostasis is, therefore, finely balanced by highly orchestrated procoagulant and anticoagulant mechanisms. Tissue factor (TF) initiated coagulation is the major mechanism that initiates thrombin generation after vascular injury. TF is a transmembrane glycoprotein expressed on the surface of various cells, particularly those surrounding blood vessels. When vascular integrity is compromised, TF is exposed to circulating plasma activated factor VII (FVIIa). TF binding allosterically activates FVIIa enabling it to proteolytically activate factor X (FX) to factor Xa (FXa) and factor IX to activated factor IX – together these steps correspond to the initiation phase of coagulation. FXa inefficiently converts small amounts of prothrombin to thrombin. Thrombin activates platelets and other coagulation procofactors, including factor VIII (FVIII) and factor V (FV). FVIIIa forms the tenase complex with activated factor IX to amplify FXa generation. After activation, FXa is released and incorporated into the prothrombinase complex together with FVa, resulting in a strong enhancement of thrombin generation and the start of the propagation phase of coagulation. Locally generated thrombin can then proteolytically convert fibrinogen into fibrin, which polymerizes to stabilize the haemostatic plug [1]. 

**Box 1:**
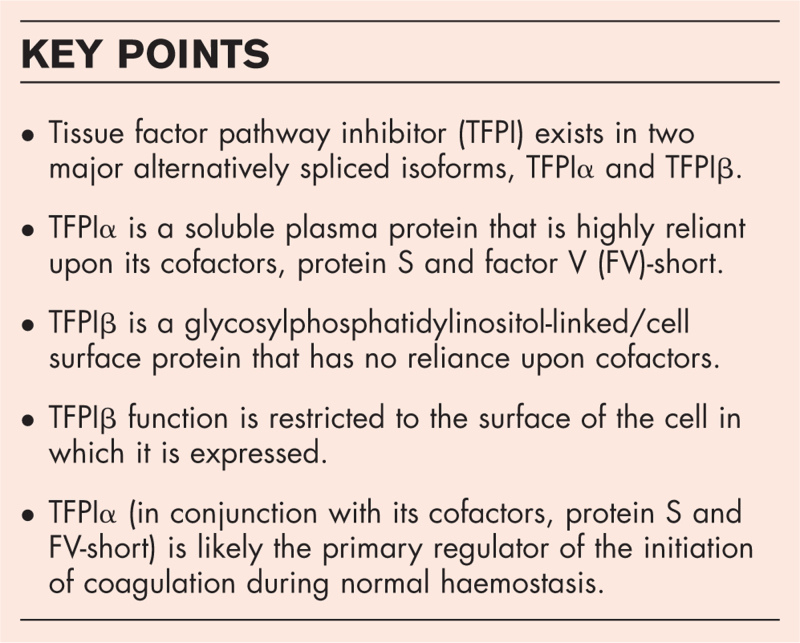
no caption available

Antithrombin, protein C and tissue factor pathway inhibitor (TFPI) are the three primary anticoagulant pathways that regulate coagulation. Antithrombin is a serpin that specifically inhibits some of the free coagulation proteases – primarily FXa and thrombin, which has led to the contention that antithrombin serves to act as a buffer that controls the active half-life of these serine proteases, as well as inhibiting the activity of these proteases if they escape the site of vessel damage [2]. Activated protein C (APC) in conjunction with its cofactor, protein S, proteolytically inactivates FVa and FVIIIa, which function in the propagation phase of coagulation, dampening further thrombin generation [3]. Given that APC is generated on the endothelial surface by thrombin-thrombomodulin (i.e. adjacent to the site of vessel damage), the suggestion is that APC may also serve to limit the spread of the haemostatic response beyond the site of injury. TFPI is a Kunitz-type protease inhibitor that specifically inhibits FXa and the TF–FVIIa complex meaning that it primarily influences the initiation of coagulation [4]. These three anticoagulant pathways therefore perform distinct, nonredundant roles that control coagulation at different times and in different locations.

**FIGURE 1:**
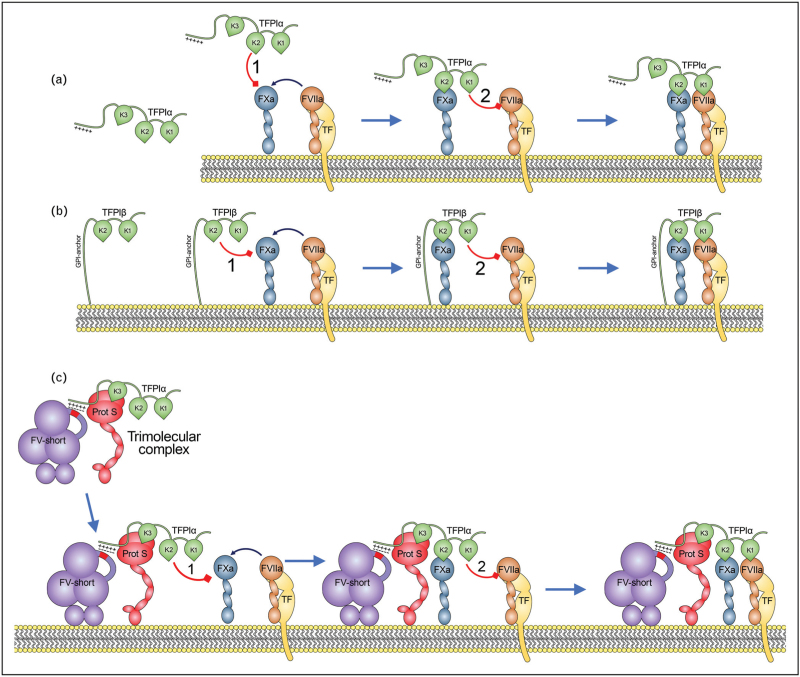
Anticoagulant function of TFPI. (a) TFPIα consists of three Kunitz domains (K1–K3) and a basic C-terminus. Following exposure of TF, the TF–FVIIa complex activates FX. TFPIα K2 inactivates FXa in a rate limiting step (1). Thereafter, TFPI–FXa inhibits TF–FVIIa to form an inactive quaternary complex. (b) The alternatively spliced TFPIβ consists of two Kunitz domains (K1–K2) coupled to the cell surface via a GPI-anchor. TFPIβ inhibits FXa and TF-FVIIa in a two-stage mechanism similar to TFPIα. (c) TFPIα forms a high affinity trimolecular complex with FV-short and protein S. TFPIα binds the acidic region of FV-short via its basic C-terminus. TFPIα and protein S do not normally interact in the absence of phospholipid surfaces. However, once bound to FV-short protein S binds with high affinity to TFPIα. This trimolecular complex has high affinity to phospholipid surfaces, which augments TFPIα anticoagulant function. GPI, glycosylphosphatidylinositol; TFPI, tissue factor pathway inhibitor; TF, tissue factor; FVII, factor VII.

In humans, TFPI exists in two major alternatively spliced isoforms, TFPIα and TFPIβ (Fig. 1a and b). TFPIα is composed of three tandem Kunitz domains (K1, K2 and K3) and a highly basic C-terminal tail [5]. K1 inhibits FVIIa in complex with TF, K2 specifically inhibits FXa, K3 is essential for interaction with its cofactor, protein S [6–8], and the basic C-terminal tail is involved with a high affinity interaction with FV-short [9]. TFPIβ is derived from the same nascent *TFPI* transcript as TFPIα, but alternative splicing gives rise to a TFPI molecule consisting of K1 and K2 that is directly attached to the surface of cells via a glycosylphosphatidylinositol (GPI) anchor [10]. The K1 and K2 domains of TFPIβ function in the same manner as those in TFPIα, but due to the lack of K3 and the basic C-terminus, TFPIβ functions independently of cofactors.

The anticoagulant function of both TFPIα and TFPIβ involves a shared two-step inhibitory mechanism (Fig. 1a and b) [11]. Initially, the K2 domain of TFPI binds to and inhibits FXa. This occurs through a rate-limiting formation of an encounter complex, followed by structural isomerization that stabilizes the inhibitory complex. The second step involves the inhibition of TF–FVIIa. TFPI alone does not inhibit TF-FVIIa. Instead, inhibition of TF-FVIIa absolutely requires prior formation of the TFPI-FXa complex. Once formed, TFPI–FXa can bind to and inhibit TF–FVIIa via the K1 domain of TFPI. This dual inhibition is essential for controlling the initiation phase of coagulation. Inhibition of FXa alone (i.e. without TF–FVIIa inhibition) is ineffective in controlling coagulation, due to the ability of TF–FVIIa to continue to generate further FXa that can rapidly overwhelm TFPI inhibitory capacity. As the primary inhibitor of TF–FVIIa, TFPI plays an essential role in not only controlling TF-dependent coagulation (i.e. ensuring a small injury does not promote an excessive coagulation response), but ultimately is also responsible for shutting down persistent *de novo* initiation of coagulation. In this way, TFPI prevents excessive thrombin generation and clot formation that can be associated with thrombotic complications.

Although TFPIα and TFPIβ share a common inhibitory mechanism, they differ appreciably in their dependence on cofactors as well as their abilities to inhibit coagulation in different locations. As a GPI-linked/cell surface protein, TFPIβ has no reliance upon cofactors but its function is restricted to the surface of the cell in which it is expressed [12]. Conversely, as a soluble plasma protein TFPIα can function on any negatively charged cell surface to which the plasma gains access. However, to do this efficiently, TFPIα is highly reliant upon its cofactors, protein S and FV-short.

Protein S, a vitamin K-dependent plasma glycoprotein, functions as a critical cofactor for TFPIα, as well as for APC. The ability of protein S to function as a cofactor in these two distinct anticoagulant pathways appears to involve some common themes. The high affinity of protein S for negatively charged phospholipid surfaces conferred by its Gla domain is central to its cofactor roles, suggesting that protein S enhances the efficiency of recruitment of either APC or TFPIα to the surfaces upon which coagulation occurs. Intriguingly, the ability of protein S to interact with either APC or TFPIα appears to be dependent on protein S first associating with phospholipid surfaces as in the absence of such surfaces protein S binds APC or TFPIα with very low affinity [6,13]. Protein S enhances TFPIα anticoagulant function in a manner that is dependent upon its laminin G-type 1 domain in the sex hormone-binding globulin (SHBG)-like region, which interacts with the K3 domain of TFPIα. Several studies have revealed that substitution/mutation of the protein S SHBG-like region or the TFPIα K3 domain diminishes protein S dependent enhancement of TFPIα [13,14^▪^,^15^]. Protein S primarily enhances the inhibitory effects of TFPIα by augmenting the rate-limiting formation of the encounter complex with FXa on phospholipid surfaces, which indirectly causes more efficient inhibition of the TF–FVIIa complex [6].

In addition to protein S, an alternatively-spliced truncated form of FV, known as FV-short exerts important anticoagulant properties by enhancing TFPIα function. Total FV plasma levels are 20–25 nM. Current estimations suggest that approximately 1–2% of FV in plasma is FV-short (0.2–0.5 nM) arising through the alternative splicing that results in the excision of the exons encoding much of the central B domain of FV [9]. In FV, the large B domain contains an acidic and a basic region, which are postulated to interact with each other [16]. In FV-short, the basic region is missing, which leaves the acidic region available [17^▪^]. This negatively charged region in FV-short along with an adjacent hydrophobic patch interacts with high affinity to the positively charged C-terminal tail of TFPIα [17^▪^,18^▪^]. The two proteins circulate as a high affinity complex in plasma that protects TFPIα from renal filtration, enabling TFPIα to persist in circulation for much longer than it otherwise would. This has led to the contention that the levels of TFPIα in plasma may be controlled by the levels of FV-short and any TFPIα that is secreted that is unable to complex with FV-short is therefore rapidly cleared. Although protein S interacts with TFPIα with low affinity in the absence of phospholipids, when in complex with FV-short, protein S appears to bind with markedly increased affinity [17^▪^,18^▪^]. This results in TFPIα circulating in a preformed trimolecular complex with both FV-short and protein S (Fig. 1c) [17^▪^,18^▪^,^19^^▪▪^]. The formation of this complex enhances TFPIα-mediated inhibition of FXa more efficiently than with protein S alone, with the two cofactors functioning in synergism. Similar to the enhancement by protein S alone, protein S together with FV-short significantly enhances the TFPIα–FXa encounter complex formation [14^▪^,^19^^▪▪^].

The precise molecular basis through which protein S and FV-short augment TFPIα function remains unclear. Originally, a working hypothesis was that the high affinity interaction of protein S for phospholipids might increase the association of TFPIα with phospholipid surfaces. This contention was based on the ∼4–10-fold enhancement of TFPI inhibition of FXa by protein S in the presence of phospholipids, but no effect in their absence [6,7,20]. However, this is complicated by the fact that TFPI and protein S only interact with very low affinity (*K*_D_ > 1 μM) in the absence of phospholipids [7,8,13,15]. This finding may be consistent with protein S undergoing a conformational change upon phospholipid binding that serves to augment TFPIα binding by several orders of magnitude (*K*_D_ ∼ 20 nM) [13,14^▪^,^19^^▪▪^]. This may be further supported by the finding that protein S can also only interact with APC in the protein C pathway following phospholipid binding. Further studies are required to probe whether such conformational changes in protein S occur and how they control its different cofactor functions.

Although protein S can interact with phospholipid surfaces and enhance recruitment of TFPIα to these surfaces, it is increasingly clear that this may not the sole mechanism involved in protein S cofactor function. The synergistic enhancement by protein S and FV-short is also completely dependent on the presence of membrane surfaces. Interestingly, FV-short binds to phospholipid surfaces with high affinity (similar to protein S), although in the absence of protein S, FV-short only very modestly enhances TFPIα inhibition of FXa [21]. This suggests that protein S fulfils additional actions (beyond recruitment to phospholipid surfaces) that augment FXa inhibition. This is supported by a recent study, showing that whilst protein S is unable to be incorporated into a TFPIα-protein S-FV-short trimolecular complex unless directly interacting with TFPIα, that same interaction is not necessary for protein S to synergistically enhance TFPIα-mediated inhibition of FXa together with FV-short [19^▪▪^]. This may potentially be mediated by a protein S-dependent structural/conformational changes on TFPI and/or FXa that promote their interaction.

The molecular basis of the effect of FV-short upon TFPIα inhibition of FXa is also rather uncertain. By itself, FV-short does not appreciably augment TFPIα inhibitory function. However, in the presence of FV-short, TFPIα can be maximally enhanced by lower concentrations of protein S [19^▪▪^]. The true physiological contribution of this effect remains unclear given that free protein S concentrations in humans (∼150 nM) would be predicted to mask this effect. However, this must not diminish the potentially highly important role of FV-short in TFPIα physiology. It may well be that the most important contribution of FV-short to TFPIα is in the control/maintenance of its plasma levels through the formation of the trimolecular complex with TFPIα and protein S, although further studies are necessary to fully define the true role of FV-short *in vivo*. This is made challenging by the appreciable differences between the human and murine TFPI systems along with the predicted absence of FV-short in mice [22].

The influence of protein S upon TFPIα anticoagulant function has been well characterized *in vitro* – primarily using FXa inhibition assays and plasma-based thrombin generation assays performed under quite specific conditions to make them maximally sensitive to the effects of TFPIα [6–8,14^▪^,^19^^▪▪^,^23^^▪▪^,^24^,^25^]. From these assays, protein S can enhance the inhibition of FXa by 4–10-fold in FXa inhibition assays. In thrombin generation assays, plasma TFPI anticoagulant function is markedly reduced in the absence of protein S [6,23^▪▪^]. Moreover, in the absence of protein S, at least fivefold higher concentrations of TFPIα are required to confer the same anticoagulant function as TFPIα in the presence of protein S [13]. Together, these data are consistent with the notion that protein S cofactor function is highly important given the very low concentrations of TFPIα in plasma (0.2–0.5 nM). However, it can be difficult to extrapolate such *in vitro* data to gauge the true physiological importance of protein S to TFPIα anticoagulant function as we do not know what the physiological ‘concentration’ range of TF after injury might be, as well as the absence of flow in *in vitro* systems that ordinarily provides a continued source of all clotting factors and inhibitors that can be otherwise depleted/consumed in a closed system. We recently endeavoured to address this issue *in vivo* using a murine laser-induced thrombosis model [23^▪▪^]. To specifically examine TFPIα anticoagulant function in mice, we blocked all endogenous murine TFPI isoforms using an antimurine K2 inhibitory antibody, which appreciably increased fibrin deposition following laser injury. Thereafter, we titrated in human TFPIα (resistant to the antimurine K2 antibody) to restore normal clot formation. Using this approach, we then specifically blocked the ability of murine protein S to function as a cofactor for human TFPIα [23^▪▪^]. These data demonstrated that, *in vivo*, TFPIα anticoagulant function is highly dependent upon its cofactor, corroborating the *in vitro* data. Moreover, a very similar high dependence of TFPIα upon human protein S was also detected using a humanized *ex vivo* microfluidic model of TF-dependent coagulation [23^▪▪^], further supporting this contention.

## CONCLUSION

To endeavour to assimilate the information above and draw conclusions, it is useful to first consider how different TFPI isoforms and different TFPI cofactors might influence haemostasis at different points. Under normal conditions TFPIα circulates at 0.2–0.5 nM in plasma, as well as within platelets. Other (truncated/lipoprotein-bound) forms of TFPI exist that circulate at higher concentrations (∼2 nM) than TFPIα, but due to the absence of the K3 domain and C-terminal tail (and therefore any cofactor enhancement), this pool appears to exert rather limited anticoagulant effect. This itself highlights the importance of cofactor-dependent enhancement in TFPIα anticoagulant function. Currently, data support the contention that FV-short levels determine the circulating steady-state levels of TFPIα. This is upheld by the elevated levels of TFPIα in individuals that have *F5* gene variations that increase the efficiency of the splicing events that generate FV-short such as those with East Texas bleeding disorder, FV-Amsterdam and FV-Atlanta [9,26,27]. These 3–10-fold elevated levels of TFPIα (linked to FV-short levels) increase bleeding risk, highlighting the anticoagulant effect of plasma TFPIα and how rather small absolute increases in TFPIα levels can cause a significant phenotypic effect. Conversely, FV deficiency is associated with decreased TFPI levels and activity [28]. Moreover, as total FV levels decrease within the normal range (likely causing a similar decrease in FV-short levels), there is perhaps a paradoxical increase risk of venous thrombosis, which may be associated with decreased levels of FV-short and the effect this has on TFPIα levels, although formal demonstration of this is still required [29]. TFPIβ is constitutively present on the endothelial surface. Under normal conditions the role of TFPIβ remains unclear given that the endothelium is not considered a source of TF in the setting of normal haemostasis. TFPIβ on the surface of other cells (particularly extravascular cells) and perhaps more importantly the TF:TFPIβ ratio may represent a means of regulating the thrombogenic potential of any given cell surface.

Following blood vessel damage, the endothelial surface is disrupted. Sub-endothelial TF-presenting cells are exposed to the blood. It seems likely that on these surfaces, the TF:TFPIβ ratio is conducive to initiation of thrombin generation. Therefore, the primary control of TF function on these cells may be more closely linked to the plasma TFPIα in a manner that is highly dependent upon its cofactors, protein S and FV-short. Under these circumstances, it is difficult to conceive a major role for endothelial TFPIβ beyond FXa inhibition given its sequestration to the surface of cells that do not express TF. During haemostatic plug formation, platelets are recruited and activated. This can result in the local release of further TFPIα from intraplatelet stores [30]. Again, the role of this pool in the setting of normal haemostasis remains unclear given that most platelets will only get activated following the initiation of coagulation (i.e. beyond the phase that TFPI can most effectively control). This pool may therefore serve to fully shut off the initiating TF stimulus to diminish prolonged *de novo* initiation of coagulation after the clot has formed. Potentially consistent with this is the recent finding that platelet TFPIα contributes to protection against cardiac thrombosis and fibrosis in mice [31^▪^].

Based on the above contentions, TFPIα (in conjunction with its cofactors, protein S and FV-short) is likely the primary regulator of the initiation of coagulation during normal haemostasis. Consistent with this TFPI plasma levels correlate with bleeding risk in patients with severe haemophilia [32^▪^]. TFPIβ on the surface of the exposed TF-presenting cells likely plays an auxiliary role during normal haemostasis. Under pathological conditions, this may of course be different. Under inflammatory conditions, such as those that may follow initiation of venous thrombosis, during severe infections or in patients with antiphospholipid syndrome, there may be conditions that can induce local expression of endothelial TF or recruitment of TF-presenting extracellular vesicles. Under such circumstances, endothelial TFPIβ may play a more prominent role in regulating the initiation of coagulation over the intact endothelium.

## Acknowledgements

*None.*

### Financial support and sponsorship

*J.T.B.C. and J.A. received research funding from a British Heart Foundation programme grant (RG/18/3/33405).*

### Conflicts of interest

*All authors report no conflicts of interest. J.A. has received research funding from AstraZeneca for work unrelated to the themes discussed herein.*

## REFERENCES AND RECOMMENDED READING

Papers of particular interest, published within the annual period of review, have been highlighted as:

▪ of special interest
▪▪ of outstanding interest
